# Blood-Stage Parasitaemia and Age Determine *Plasmodium falciparum* and *P*. *vivax* Gametocytaemia in Papua New Guinea

**DOI:** 10.1371/journal.pone.0126747

**Published:** 2015-05-21

**Authors:** Cristian Koepfli, Leanne J. Robinson, Patricia Rarau, Mary Salib, Naomi Sambale, Rahel Wampfler, Inoni Betuela, Wang Nuitragool, Alyssa E. Barry, Peter Siba, Ingrid Felger, Ivo Mueller

**Affiliations:** 1 Walter and Eliza Hall Institute, Population Health and Immunity Division, Parkville, Victoria, Australia; 2 University of Melbourne, Department of Medical Biology, Parkville, Victoria, Australia; 3 Papua New Guinea Institute of Medical Research, Vector Borne Diseases Unit, Madang, Papua New Guinea; 4 Swiss Tropical and Public Health Institute, Medical Parasitology and Infection Biology, Basel, Switzerland; 5 University of Basel, Basel, Switzerland; 6 Faculty of Tropical Medicine, Mahidol University, Bangkok, Thailand; 7 ISGlobal, Barcelona Centre for International Health Research, Barcelona, Spain; Centro de Pesquisa Rene Rachou/Fundação Oswaldo Cruz (Fiocruz-Minas), BRAZIL

## Abstract

A better understanding of human-to-mosquito transmission is crucial to control malaria. In order to assess factors associated with gametocyte carriage, 2083 samples were collected in a cross-sectional survey in Papua New Guinea. *Plasmodium* species were detected by light microscopy and qPCR and gametocytes by detection of *pfs25* and *pvs25 *mRNA transcripts by reverse-transcriptase PCR (qRT-PCR). The parasite prevalence by PCR was 18.5% for *Plasmodium falciparum *and 13.0% for *P*. *vivax*. 52.5% of all infections were submicroscopic. Gametocytes were detected in 60% of *P*. *falciparum*-positive and 51% of *P*. *vivax*-positive samples. Each 10-fold increase in parasite density led to a 1.8-fold and 3.3-fold increase in the odds of carrying *P*. *falciparum* and *P*. *vivax* gametocytes. Thus the proportion of gametocyte positive and gametocyte densities was highest in young children carrying high asexual parasite densities and in symptomatic individuals. Dilution series of gametocytes allowed absolute quantification of gametocyte densities by qRT-PCR and showed that *pvs25 *expression is 10-20 fold lower than *pfs25 *expression. Between 2006 and 2010 parasite prevalence in the study site has decreased by half. 90% of the remaining infections were asymptomatic and likely constitute an important reservoir of transmission. However, mean gametocyte densities were low (approx. 1-2 gametocyte/μL) and it remains to be determined to what extent low-density gametocyte positive individuals are infective to mosquitos.

## Introduction

Understanding *Plasmodium spp*. transmission from the human host to the mosquito vector is a key priority in the attempt to control and eventually eliminate malaria. While most malaria parasites in the human host replicate asexually, a small proportion of them enter the sexual pathway and form morphologically and functionally different gametocytes. Malaria transmission depends on mosquitos taking up both male and female gametocytes and transmitting their progeny to the next human host.

The biology of *Plasmodium falciparum* and *P*. *vivax* gametocytes differ [1]. *P*. *falciparum* gametocytes are sequestered during development and only mature gametocytes circulate. They appear 7–15 days after the initial wave of asexual stages in the blood stream [2], thus usually after the first febrile symptoms. In contrast *P*. *vivax* gametocytes appear in the blood stream shortly after the first appearance of asexual blood stage parasites and before the onset of clinical disease [3]. In addition individual *P*. *falciparum* gametocytes circulate for 3–4 weeks [4,5], while *P*. *vivax* gametocytes only survive for a few days [6].

By light microscopy (LM), the proportion *P*. *falciparum* gametocyte positive (i.e. the proportion of gametocyte carriers among all *P*. *falciparum* positive individuals) in symptomatic individuals with high asexual parasite densities (often >5000 parasites/μL) ranged from 2.4 to 17% in Thailand and West Africa [7–11]. When gametocyte carriage was assessed by LM in both *P*. *falciparum* and *P*. *vivax* clinical cases, the proportion *P*. *vivax* gametocyte positive was 6–8 times higher than that of *P*. *falciparum* [8,12,13] reaching rates of 57–92% in different Asian countries [8,14,15]. The prevalence of gametocytes in the general population, in particular in asymptomatic individuals, has been studied in only few malaria-endemic regions [16–18]. A study in Madang Province, Papua New Guinea reported that 9.8% of *P*. *falciparum-* compared to 36.3% of *P*. *vivax*-infected individuals carried gametocytes [19]. The proportion gametocyte positive was highest in children and decreased rapidly with age.

As the amount of blood examined is limited and only a small fraction of all parasites are gametocytes, microscopic detection of gametocytes has limited sensitivity of around 10–20 gametocytes/μL blood. Molecular assays, based on amplification of gametocyte specific RNA transcripts, have revealed 2–8 times higher gametocyte prevalence than light microscopy [20–24]. Using molecular assays, proportions of *P*. *falciparum* gametocyte carriers amongst *P*. *falciparum* positive individuals ranged from 12% in asymptomatic carriers [25] to 15% in a cross-sectional survey [20] and 89–91% in clinical infections [21,24]. The proportion *P*. *vivax* gametocyte positive reached 95% both in febrile patients [24] and in cross-sectional surveys [26].

In mosquito feeding experiments it has been repeatedly shown that individuals that carry gametocytes at submicroscopic levels are infective, both for *P*. *falciparum* [23,27,28] and *P*. *vivax* [29,30]. While individuals who were gametocyte positive by LM were considerably more infective than individuals with only asexual parasites [13,28,30,31], the latter likely contribute substantially to transmission due to the large proportion of asymptomatic individuals carrying low-moderate density infections, [23].

Little is known about the factors associated with gametocyte prevalence and density, such as acquired immunity, clinical disease and age. To identify carriers of *P*. *falciparum* and *P*. *vivax* gametocytes, we conducted a cross-sectional survey in Papua New Guinea. Blood-stage parasites and gametocytes were detected by highly sensitive quantitative PCR (qPCR) and reverse-transcriptase qPCR (qRT-PCR) assays.

## Material and Methods

### Ethical statement

Written informed consent was obtained from all study participants or their parents or legal guardians. The study was approved by PNG IMR IRB, the PNG Medical Research Advisory Committee (MRAC 05.20 & 12.06) and the WEHI Human Research Ethics Committee.

### Sample collection

The study was conducted in May-June 2010 in 3 communities (Malala, Mugil and Utu) in Madang Province PNG, approximately two years after long-lasting insecticide treated bed nets (LLINs) were distributed to all households [32]. The region receives over 3,000 mm of rainfall annually with a short dry season (June to October) and has hyper-endemic malaria transmission that is moderately seasonal (lower in the drier season).

From each participant demographic data was recorded, history of febrile illness and data on bed net use. Axillary temperature was measured, 250–300μL of capillary blood collected into a K+EDTA microtainers, thick/thin films were prepared and haemoglobin measured (Hemocue). For statistical purposes individuals were classified as non-anaemic (Hb ≥11g/dL), having mild anaemia (Hb 8–10.9 g/dL), and having moderate-to-severe anaemia (Hb <8g/dL). In the case of reported febrile illness or axillary temperature >37.5°C, a rapid malaria diagnostic test (ICT Combo) was performed and those positive by RDT treated with Artemether-Lumefantrine (Coartem). For RNA preservation, 50μL of whole blood was transferred to a tube containing 250μL of RNAProtect (Qiagen) within 8 hours of collection and stored at -80°C until RNA extraction. In previous studies, transfer of blood to RNAprotect within 4 hours after collection was shown to maintain high-quality RNA and thus optimal sensitivity of gametocyte detection [33]. In addition, no effect on RNA quality was observed when samples were stored at ambient temperature for up to 24 hours prior to long-term storage at -80° (A. Waltmann, S. Karl *et al*, manuscript in preparation). The remaining blood was centrifuged, the plasma removed and stored at -80°C, and the red cell pellet stored at -20°C until DNA extraction.

### Parasite detection

Blood slides were examined (minimum of 200 high-powered fields) independently by two experienced microscopists with discrepancies adjudicated by a third microscopist. Parasite densities were calculated from the number of parasites per 200 or 500 white blood cells (WBCs) (depending on parasitaemia) and an assumed total peripheral WBC count of 8,000/μL [34] with the final density taken as the arithmetic mean of the two values.

DNA was extracted from the equivalent of 200μL blood using the Favorgen 96-well Genomic DNA Extraction Kit and eluted in 200μL buffer. A genus-specific quantitative PCR (qPCR) that amplifies a conserved region of the 18S rRNA gene was run on all samples [33]. For *P*. *falciparum*, *P*. *vivax*, *P*. *malariae* and *P*. *ovale* species typing, species-specific qPCRs were run as described [35]. The 18S rRNA gene amplified by qPCR is present in 3 copies per genome. Copy numbers were quantified based on serial dilutions of plasmid controls run in parallel. The detection limit (defined as >50% of plasmid standards positive) was 1–2 copies per μL for all qPCRs.

### Gametocyte detection and quantification

The number of *P*. *falciparum* and *P*. *vivax* gametocytes were recorded separately from asexual stages during microscopic examination of all blood slides.

For gametocyte detection by qRT-PCR, RNA was extracted from all *P*. *falciparum* or *P*. *vivax* qPCR-positive samples using the Qiagen RNeasy plus 96 kit, including Genomic DNA removal by gDNA eliminator columns and DNase (Qiagen). Absence of gDNA in all samples was confirmed by the same genus-specific, DNA-based qPCR used for parasite detection. In a separate PCR reaction, presence of parasite RNA after extraction was verified by quantitative reverse-transcriptase PCR (qRT-PCR) detecting 18S-RNA with the same primers and probe as the genus-specific qPCRs.

*P*. *falciparum* and *P*. *vivax* gametocytes were detected by qRT-PCR of the highly expressed gametocyte markers *pfs25* and *pvs25* as described [33]. The number of *pfs25* transcripts per *P*. *falciparum* gametocyte had previously been calculated based on comparisons of microscopy counts and qRT-PCR results of different dilutions of cultured *P*. *falciparum* gametocytes. A non-linear relation was found and it was estimated that the qRT-PCR detects 50–180 *pfs25* transcripts per circulating *P*. *falciparum* gametocyte, depending on gametocyte density [33].

Two independent *P*. *vivax* gametocyte trend-lines were generated from enriched gametocytes from *P*. *vivax* patients from Thailand (2 isolates, 21–24% gametocytes, 64–70% of all gametocytes female) and Brazil (3 isolates, 23–42% gametocytes, 24–71% of all gametocytes female). Leukocyte-depleted blood was passed through a MidiMacs column (Miltenyi Biotec), gametocytes and other stages (mostly trophozoites) were eluted and resuspended in RPMI medium. Two samples were stored in TRIzol, and a 10-fold serial dilution (5 steps) was made after RNA extraction. Three samples were diluted 10-fold (3 steps) before adding RNAprotect and storing. The final RNA concentration corresponded to densities of 10^4^ to 1 gametocytes/μL blood. *Pvs25* transcripts/μL were quantified by qRT-PCR; plasmid standards were run in parallel for absolute quantification. Conversion factors for *pvs25* transcript numbers into numbers of gametocytes were calculated with a random-effect model from log10-transformed quantities of the gametocyte trend-lines. Samples were set as random effect.

### Data analysis

Malaria episodes were defined as febrile illness (axillary temperature >37.5°C measured at time of sampling and/or fever reported from previous 48 hours) in the presence of *P*. *vivax* or *P*. *falciparum* asexual parasites by LM (any density). Parasite and gametocyte densities were log transformed and geometric means per μL whole blood calculated wherever densities are reported. Multivariate analysis of factors associated with gametocyte carriage was conducted using logistic regression and associations with gametocyte density investigated by linear regression. All analyses were performed using Stata 12 or R version 2.14.0.

## Results

A total of 2083 individuals with age distribution representative for the population were surveyed; demographic and clinical characteristics are summarized in Table 1.

**Table 1:** Demographic, clinical and parasitological characteristics of study participants.

		n	%
		2083	100
**Area**	Malala	936	44.9
	Mugil	799	38.4
	Utu	348	16.7
**Age group (n = 1967)**	0–3	291	14.0
	>3–6	228	10.9
	>6–9	224	10.6
	>9–12	175	8.5
	>12–20	198	9.5
	>20	851	40.9
**Bednet use (n = 2065)**	Yes	1594	76.5
**Malaria last 2 weeks (n = 2973)**	Yes	166	8.0
**Anti-malarials in last 2 weeks (n = 2078)**	Yes	61	2.9
**Anaemia (n = 1816)**	No	763	36.6
	Mild	942	45.2
	Moderate	111	5.3
**Current or recent febrile illness (n = 2083)**	Yes	199	9.5
**Clinical Malaria**	*P*. *falciparum*	21	1.0
	*P*. *vivax*	9	0.4
	*Pf*/*Pv* mixed	5	0.2
***Plasmodium spp*. infection by LM**	*P*. *falciparum*	155	7.4
	asexuals only	82	
	gametocytes only	38	
	both	35	
	*P*. *vivax*	146	7.0
	asexuals only	103	
	gametocytes only	3	
	both	40	
	*P*. *malariae*	6	0.3
	*P*. *ovale*	0	0
	*Pf*/*Pv* mixed	10	0.9

Overall, the prevalence rate (PR) by light microscopy was 7.4% *P*. *falciparum*, 7.0% *P*. *vivax*, 0.29% *P*. *malariae*, 0% *P*. *ovale and* 0.9% mixed *P*. *falciparum/P*. *vivax*. The overall prevalence of clinical malaria was 1.6% (n = 34); 88.1% of all LM-positive infections were asymptomatic (Table 1).

**Fig 1:**
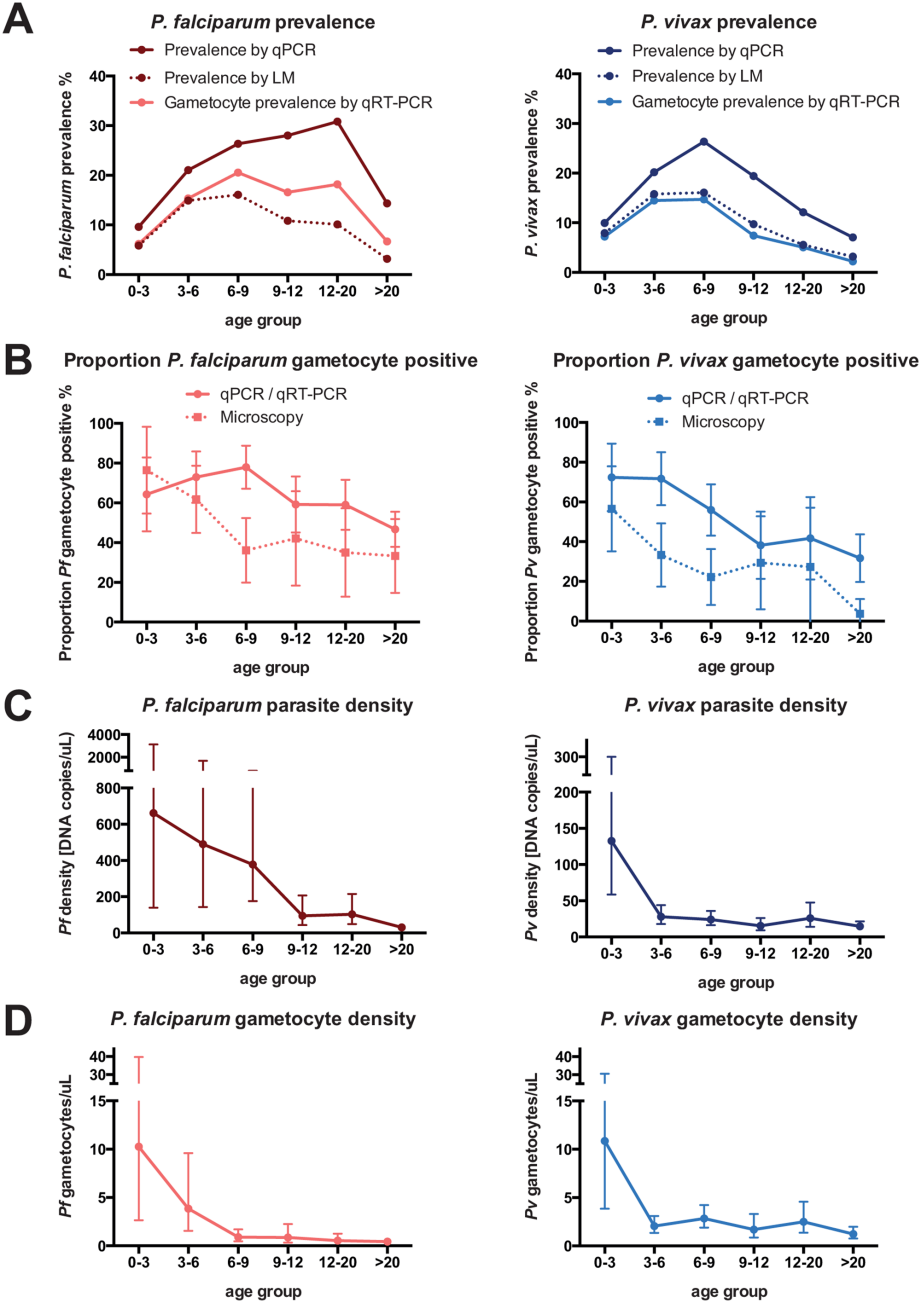
Age trends in *P*. *falciparum* and *P*. *vivax* blood-stage and gametocyte prevalence and density. Error bars represent CI_95_.

52.9% of *P*. *falciparum*-positive samples had only asexual stages, 24.5% only gametocytes and 22.6% both. Of *P*. *vivax*-positive samples 70.5% had asexual stages only, 2.1% gametocytes only and 27.4% both. By LM *P*. *falciparum* gametocytes were thus detected in 73 (3.5%) individuals and *P*. *vivax* gametocytes in 43 (2.1%) individuals. Geometric mean asexual and sexual parasite densities by LM were 808.0 (CI_95_[579.3–1127.0]) and 158.3 (CI_95_[117.4–213.3]) *P*. *falciparum* parasites/μL and 117.6 (CI_95_[97.3–142.1]) and 34.9 (CI_95_[26.9–45.3]) *P*. *vivax* parasites/μL. For both species, overall prevalence (Fig 1A, *P*<0.001) and the proportion gametocyte positive (Fig 1B, *Pf*: *P* = 0.010, *Pv*: *P* = 0.002) differed significantly between age groups.

### Parasite prevalence by PCR

By qPCR, 385 out of 2083 samples (18.5%) were positive for *P*. *falciparum* and 270 (13.0%) for *P*. *vivax*. Of these, 81 (3.9%) carried both species. *P*. *malariae* and *P*. *ovale* were rare (28 and 2 infections) and occurred mostly (17/30) in participants concurrently infected with *P*. *falciparum* and/or *P*. *vivax*. Of all individuals positive by PCR, 52.5% were negative by LM, 89.6% were asymptomatic and 5.6% met the definition of clinical malaria (fever and parasites detected by LM). Prevalence for *P*. *falciparum* and *P*. *vivax* showed pronounced differences between age groups (Fig 1A, *P*<0.001) with *P*. *falciparum* peaking in individuals 12–20 years of age and *P*. *vivax* peaking in children 6–9 years of age. *P*. *falciparum* prevalence was higher in mild-moderately anaemic and febrile individuals, whereas *P*. *vivax* was less common in individuals with recent malaria episodes (Tables 2 and 3).

**Table 2:** Multivariate predictors of *P*. *falciparum* parasite prevalence and proportion of gametocyte positive.

		*Parasite prevalence* (n = 1,714)				*Proportion gametocyte positive* (n = 349)			
		%	aOR	95% CI	*P*-value	%	aOR	95% CI	*P*-value
**Gene copies/μL (log)** ^1^							1.85	[1.50, 2.28]	<0.001
**Mixed Pf/Pv (by pcr)**	No					63.8	1		
	Yes					48.2	0.40	[0.23, 0.71]	0.002
**Age group**	0–3	9.6	1		<0.001	64.3			
	>3–6	21.1	2.34	[1.38, 3.97]		72.9			
	>6–9	26.3	3.40	[2.04, 5.68]		78.0			
	>9–12	28.0	3.65	[2.12, 6.29]		59.2			
	>12–20	30.8	4.87	[2.88, 8.24]		59.0			
	>20	14.3	1.78	[1.13, 2.81]		46.7			
**Malaria last 2 weeks**	No	18.1				57.7	1		
	Yes	22.3				83.8	3.78	[1.42, 10.1]	0.008
**Febrile**	No	17.8	1		0.001	58.0			
	Yes	24.6	1.87	[1.27, 2.75]		77.6			
**Anaemia**	No	14.7	1		<0.001	49.1	1		0.019
	Mild	23.0	1.78	[1.36, 2.34]		66.1	1.63	[0.99, 2.70]	
	Moderate	20.7	1.70	[1.01, 2.84]		52.2	0.46	[0.16, 1.33]	

aOR = adjusted odds ratio (adjusted for multivariate analysis), CI = confidence interval

^**1**^ Increase in the probability that gametocytes are detected in a *P*. *falciparum* positive sample, if the parasite density increases 10-fold

**Table 3:** Multivariate predictors of *P*. *vivax* parasite prevalence and proportion gametocyte positive.

		Parasite prevalence (n = 1,958)				Proportion gametocyte positive (n = 224)			
		%	aOR	95% CI	*P*-value	%	aOR	95% CI	*P*-value
**Gene copies/μL (log)** ^**1**^							2.85	[1.74, 4.67]	<0.001
**Mixed Pf/Pv (by pcr)**	No					48.7			
	Yes					49.4			
**Age group**	0–3	10.0	1		<0.001	72.4	1		0.011
	>3–6	20.2	2.35	[1.41, 3.90]		71.7	1.10	[0.32, 3.75]	
	>6–9	26.3	3.30	[2.02, 5.40]		55.9	0.53	[0.17, 1.68]	
	>9–12	19.4	2.26	[1.31, 3.88]		38.2	0.36	[0.10, 1.33]	
	>12–20	12.1	1.26	[0.71, 2.25]		41.7	0.33	[0.08, 1.31]	
	>20	7.0	0.69	[0.43, 1.11]		31.7	0.20	[0.06, 0.67]	
**Malaria last 2 weeks**	No	13.5			0.029	49.2			
	Yes	6.0	0.48	[0.25, 0.93]		40.0			
**Febrile**	No	13.2				48.8			
	Yes	11.1				50.0			
**Anaemia**	No	11.0				41.7	1		0.048
	Mild	15.5				47.3	0.66	[0.34, 1.30]	
	Moderate	9.0				90.0	8.56	[0.94, 77.7]	

aOR = adjusted odds ratio (adjusted for multivariate analysis), CI = confidence interval

^1^ Increase in the probability that gametocytes are detected in a *P*. *vivax* positive sample, if the parasite density increases 10-fold

Geometric mean parasite densities by qPCR were very low with 126.8 (CI_95_ [91.1–176.5]) copies per μL/blood for *P*. *falciparum* and 23.8 (CI_95_ [19.5–29.1]) copies/μL for *P*. *vivax*. Assuming that all 3 copies of the marker gene were amplified, this corresponds to 42 *P*. *falciparum* and 8 *P*. *vivax* genomes per μL. Densities of *P*. *falciparum* and *P*. *vivax* were 14- and 10-fold lower in adults as compared to children <3 years (Fig 1C), and *P*. *vivax* densities dropped more rapidly with age than *P*. *falciparum* densities (Fig 1C). In samples that were positive by both qPCR and LM, densities were more highly correlated for *P*. *falciparum* (*r*_S_ = 0.81, n = 144) than *P*. *vivax* (*r*_S_ = 0.39, n = 139, S1 Fig).

### Gametocyte prevalence by qRT-PCR

By qRT-PCR, 233/385 (60.5%) of *P*. *falciparum* positive and 132/270 (48.9%) of *P*. *vivax* positive individuals carried detectable gametocytes, resulting in a population gametocyte prevalence of 11.2% and 6.3%.

**Fig 2:**
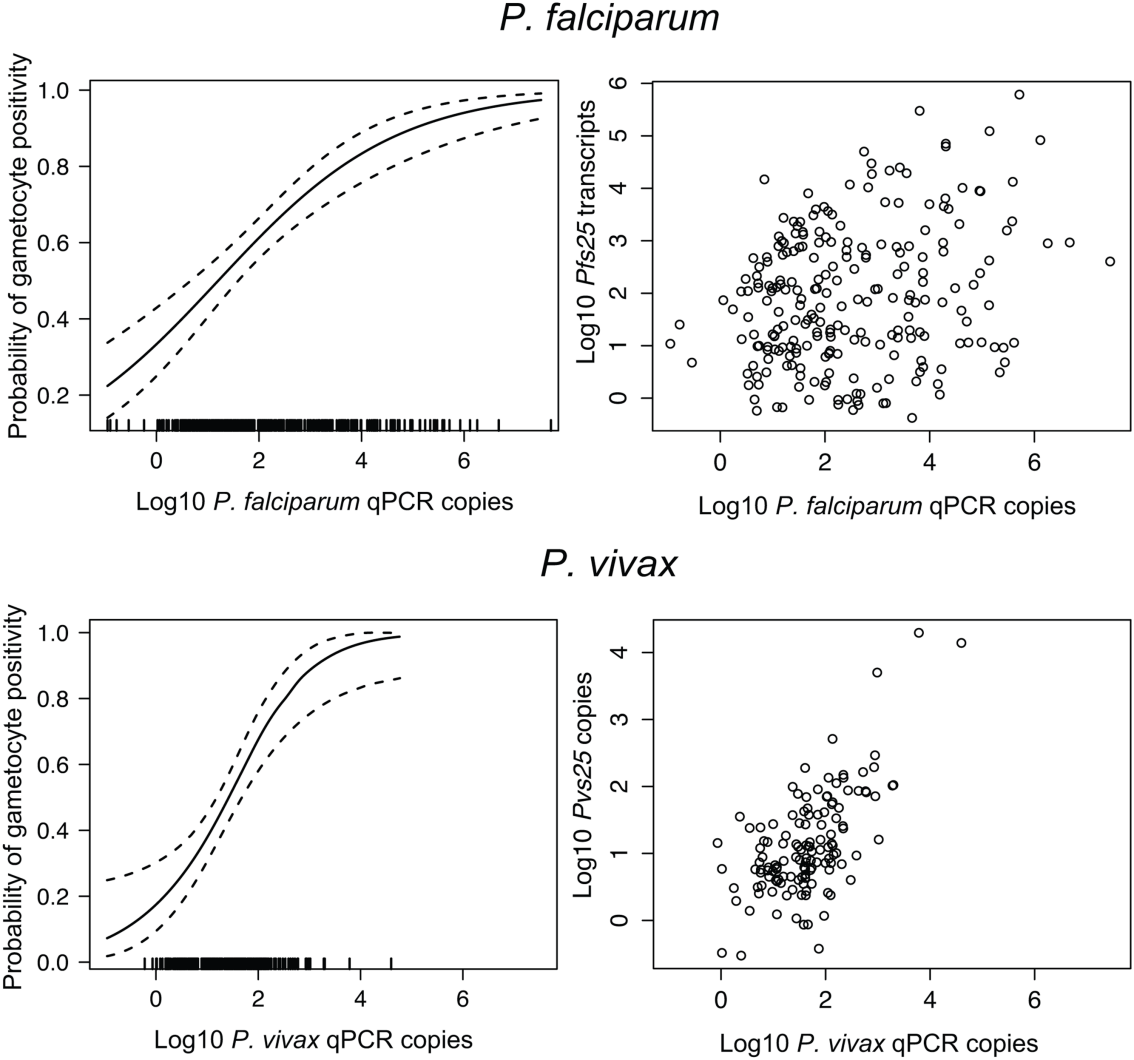
*pvs25* transcript numbers versus light microscopically determined *P*. *vivax* gametocyte counts. Dilution series were made before storing samples in RNAprotect (5 dilution steps) or after RNA extraction of samples stored in TRIzol (3 dilution steps). Dots represent means of technical triplicates (TRIzol) or quintuplicates (RNAprotect) of dilutions except for the highest concentration (no replicates). Shaded grey shows the CI_95_ of the intercept of the regression of compiled sample sets.

For both species, the likelihood to detect gametocytes was considerably higher in samples with high blood-stage parasitaemia (Fig 2). Each log increase in parasite genome copy density was associated with a 1.77-fold increase (CI_95_[1.47–2.13], *P*<0.001) in the odds of carrying *P*. *falciparum* and a 3.27-fold increase (CI_95_[2.16–4.95], *P*<0.001) in the odds of carrying *P*. *vivax* gametocytes. As a result gametocytes were detected in nearly all LM-positive infections (*Pf*: 88.2%, *Pv*: 79.3%) but in a much lower proportion of submicroscopic infections (*Pf*: 44.0%, *Pv*: 16.1%, *P*<0.001). *P*. *falciparum* gametocyte densities were 6-fold higher in samples positive by microscopy as compared to submicroscopic infections (2.45 [1.50–3.99] vs. 0.39 [0.28–0.56] gametocytes/uL), while *P*. *vivax* gametocyte densities were similar in both groups (2.77 [2.07–3.71] vs. 2.22 [1.37–3.61] gametocytes/uL). Co-infections with *P*. *vivax* resulted in a significantly lower proportion *P*. *falciparum* gametocyte positive (48.1% vs. 63.8%, *P* = 0.010) but not vice versa.

*P*. *falciparum* gametocyte prevalence and the proportion gametocyte positive peaked in children 6–9 years of age (20.5% and 78.0%) and was lowest (6.7% and 46.7%) in adults (*P* = 0.001, Fig 1, Table 2). *P*. *vivax* gametocyte prevalence among all study participants decreased from 14.6% in children 3–9 years to 2.2% in adults (*P*<0.001; Table 3) while the proportion *P*. *vivax* gametocyte positive dropped from 71–72% in children below 6 years to 31.7% in adults.

The proportion gametocyte positive for both species was also associated with haemoglobin levels, albeit in different ways. *P*. *falciparum* gametocytes were most commonly present in participants with mild anaemia (Table 2, Hb 8.0–10.9g/dL, 66.0%, *P* = 0.008), whereas *P*. *vivax* gametocytes were most common in participants with moderate-to-severe anaemia (Hb <8g/dL; Table 3, 90.0%). Lastly, participants who reported a malaria episode in the last 2 weeks were more likely to have *P*. *falciparum* gametocytes (83.8% vs. 57.7%, *P* = 0.002; Table 2). After accounting for all other factors, blood-stage parasitaemia remains the most important predictor of carriage of gametocytes (Tables 2 and 3).

All symptomatic *P*. *falciparum* and 11/13 *P*. *vivax* infections carried gametocytes. However, when corrected for asexual densities clinical malaria itself was not significantly associated with gametocyte carriage for either species. Blood-stage *P*. *falciparum* and *P*. *vivax* parasitaemia was 570- and 5-fold higher in symptomatic than in asymptomatic individuals (*Pf*: 48212 CI_95_[14358–161888] vs. 84.0 CI_95_ [62.1–113.6] parasites/μL, *Pv*: 104.4 CI_95_[28.2–387.1] vs. 21.8 CI_95_[17.9–26.6] parasites/μL).

### Quantification of gametocytes by qRT-PCR

**Fig 3:**
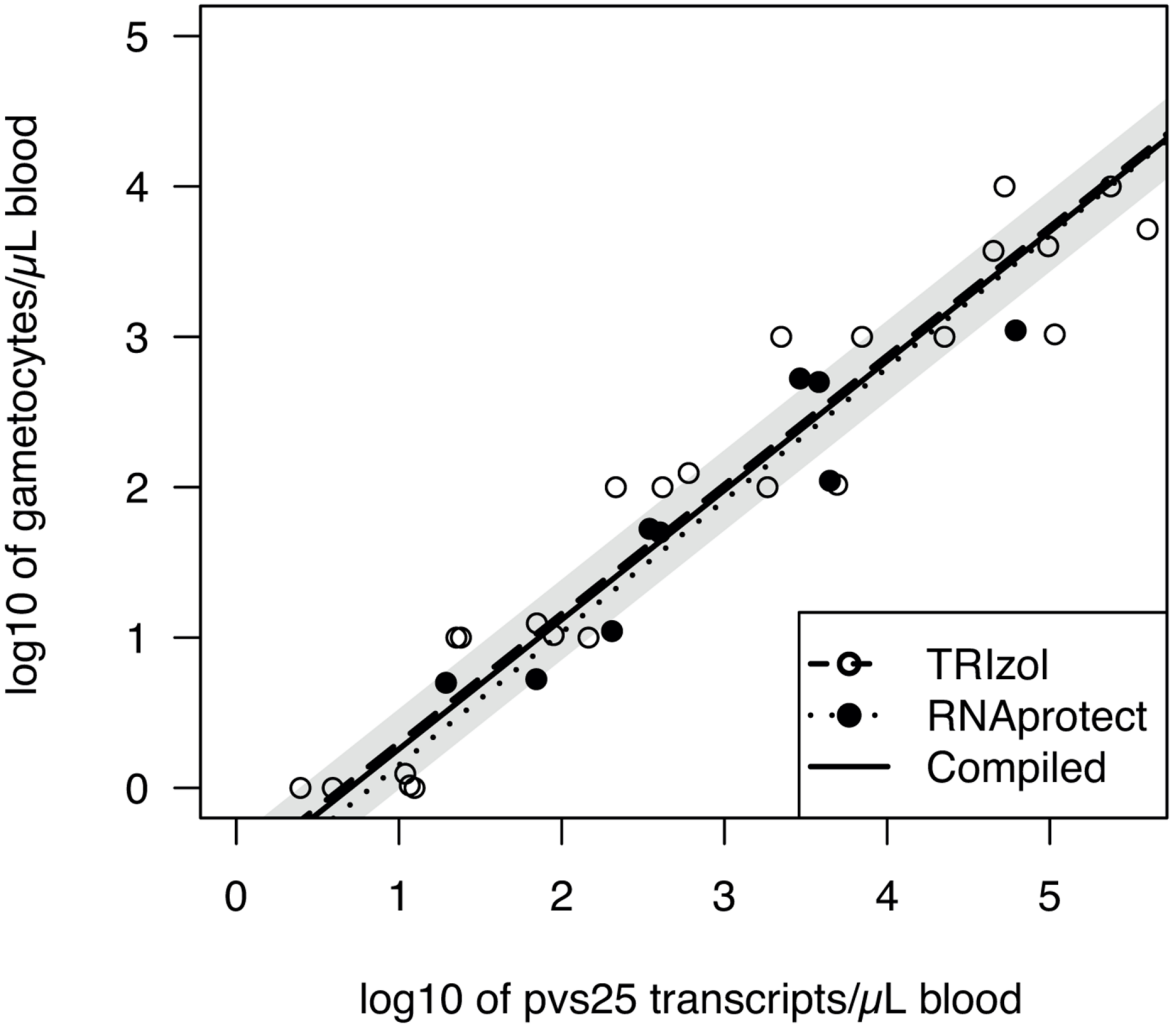
Relationship between parasite density and probability that a sample is gametocyte positive. Left: Probability (with CI_95_) that a sample is gametocyte positive vs. parasite density, calculated using a general additive model. Right: correlation between gametocyte density (measured as *pfs25* or *pvs25* transcripts/uL) and parasite density by qPCR.

*pfs25* densities were converted to *P*. *falciparum* gametocyte densities based on published trend-lines: gametocytes/μL = 10^−1.6225^*t_*pfs25*_^0.8518^, where t_*pfs25*_ is the number of *pfs25* transcripts/μL detected by qRT-PCR [33]. This corresponds to approximately 50–180 *pfs25* transcripts per cultured *P*. *falciparum* gametocyte. For *pvs25* expression, storage in TRIzol or RNAprotect resulted in very similar trend-lines (Fig 3). The conversion [with CI_95_] obtained from samples stored in TRIzol was: gametocytes/μL = 10^−0.5546[-0.8973;-0.2120]^*t_*pvs25*_^0.8576[0.8057;0.9095]^. Conversion of samples stored in RNAprotect was: gametocytes/μl = 10^−0.7283[-1.3802; -0.0765]^*t_*pvs25*_^0.8804[0.7041; 1.0567]^. For all further analysis, data was pooled and the following conversion applied: gametocytes/μL = 10^−0.5171[-0.7452;-0.2890]^*t_*pvs25*_^0.8339[0.8033;0.8645]^. Thus one gametocyte roughly corresponds to 4.17 [2.16–8.47] *pvs25* transcripts, though this is not a linear, but a logarithmic relationship, where higher gametocyte densities show a higher number of transcripts/gametocyte.

Among qRT-PCR positive participants, mean *pfs25* density was 86.4 transcripts/μL (CI_95_[58.7–127.1]), and mean *pvs25* density was 13.6 transcripts/μL (CI_95_[10.0–18.4]). This corresponds to 1.06 (CI_95_[0.77–1.48]) *P*. *falciparum* and 2.67 (CI_95_[2.07–3.45]) *P*. *vivax* gametocytes/μL. Gametocyte densities were significantly higher in participants with LM-detectable gametocytaemia (*Pf*: 12.1 vs. 0.37/μL *P*<0.001; *Pv*: 6.5 vs. 1.75/μL, *P*<0.01). For samples with ≥1 gametocyte/μL by qRT-PCR, a strong correlation between densities by LM and qRT-PCR was observed (*Pf*: *r* = 0.696, *P*<0.001, *Pv*: *r* = 0.574, *P* = 0.001, for log-transformed values).

*P*. *vivax* gametocyte densities were strongly correlated with *P*. *vivax* blood-stage parasitaemia by qPCR (*r* = 0.636, *P*<0.001, log-transformed values). A small but still highly significant correlation was also found between *P*. *falciparum* gametocyte and genome copy numbers (*r* = 0.259, *P*<0.001) (Fig 2).

The age-associated reduction in gametocyte densities was even faster than that for blood-stage parasite densities (Fig 1D, *P*<0.001). *P*. *falciparum* and *P*. *vivax* gametocyte densities dropped 2.7-fold and 5.3-fold between children 0–3 and 3–6 years of age. *P*. *falciparum* gametocyte densities were significantly higher in individuals who reported a recent malaria episode (5.7 vs. 0.83 gametocytes/μL *P*<0.001). These factors remain significantly associated in multivariate analyses (Table 4).

**Table 4:** Determinants of *P*. *falciparum* and *P*. *vivax* gametocyte density.

		*P*. *falciparum*				*P*. *vivax*			
		GMD	β	95% CI	*P*-value	GMD	β	95% CI	*P*-value
**Gene copies/μL (log)**			0.13	[0.02, 0.24]	0.022		0.57	[0.43, 0.72]	<0.001
**Age group**	0–3	21.1	0		<0.001	10.5	0		0.026
	>3–6	15.2	-0.46	[-1.14, 0.21]		2.8	-0.47	[-0.81, -0.13]	
	>6–9	2.8	-1.04	[-1.70, -0.39]		2.0	-0.23	[-0.57, 0.11]	
	>9–12	8.9	-1.06	[-1.77, -0.35]		1.8	-0.55	[-0.97, -0.13]	
	>12–20	5.0	-1.31	[-2.00, -0.63]		1.9	-0.45	[-0.90, -0.00]	
	>20	3.2	-1.39	[-2.05, -0.74]		1.9	-0.55	[-0.95, -0.16]	
**Malaria last 2 weeks**	Yes	28.3	0.94	[0.48, 1.40]	<0.001	61.9			NS
	No	4.3				2.5			
Intercept			0.53	[-0.15, 1.20]			-0.46	[-0.88, -0.04]	0.032

GMD = geometric mean density, β = regression coefficient, CI = confidence interval

## Discussion

Mass-distribution of LLINs in 2008 resulted in ~70% coverage [32] and led to a substantial reduction in malaria transmission in the study area. Compared to 2006 the prevalence of *P*. *falciparum* and *P*. *vivax* infections dropped from 39% to 18% and from 32% to 13%, respectively [36,37]. Considerably fewer mixed-species infections were detected as compared to previous studies in PNG, where up to 32% of all individuals sampled were infected by several species concurrently [38,39]. Nevertheless, transmission of *P*. *falciparum* and *P*. *vivax* on the north coast of PNG remains high, clinical immunity is acquired rapidly and—as a consequence—the vast majority of infections are asymptomatic (90%) and of low density (52% submicroscopic). These infections are not targeted by the current control strategy of ACT-based case management plus LLINs, and thus constitute a potentially important reservoir of transmission.

qRT-PCR detected gametocytes in 60% of *P*. *falciparum* and 51% of *P*. *vivax* positive individuals. The results corresponded very well with an earlier PNG study that used identical methods and found 59% of *P*. *falciparum* and 52% of *P*. *vivax* positive children aged 5–10 carrying gametocytes [33]. Gametocyte carriage was low compared to other studies, where nearly all infected individuals carried gametocytes [22–24,26] and contradicts earlier studies based on light microscopy that reported that considerably higher proportions of *P*. *vivax* than *P*. *falciparum* infections carried gametocytes [8,13]. However, most of these other studies were conducted on symptomatic patients with relatively high parasitaemia (>5000 parasites/μL) [8,13,22,24,26].

In this cross-sectional study, blood-stage parasite density was the strongest predictor for gametocyte carriage. Thus for both species gametocytes were detected in almost all symptomatic and the vast majority of LM-positive infections.

Parasite densities decrease at an early age, because in areas of intense transmission many individuals are immune or semi-immune to malaria [38,39]. Thus, the proportion of gametocyte positive individuals was highest in young children. As only a small fraction of all parasites were gametocytes, the number of gametocyte carriers detected among individuals carrying low parasite densities was heavily dependent on the sensitivity of the assays and the amount of blood assessed. Both the *pfs25* and *pvs25* qRT-PCRs were able to detect as little as 1–2 transcripts/μL blood. Using the same detection techniques for *P*. *falciparum* gametocytes from culture [33] and serial dilutions of *P*. *vivax* gametocytes from clinical cases, around 10-times more *pfs25* than *pvs25* transcripts/gametocyte were detected. Thus in field samples as little as a single *P*. *falciparum* gametocyte can be detected in 50μL blood, assuming it provides 1–2 *pfs25* transcripts/μL extracted RNA. For *P*. *vivax*, however, a density of 10 gametocytes/50μL blood would be required for an equal probability of detection. This explains why no gametocytes were detected in 84% of all submicroscopic *P*. *vivax* infections, and where detected, their similar densities in microscopy positive and submicroscopic infections. Likely gametocytes were present in some of the submicroscopic, gametocyte negative infections, yet at densities below the detection limit of qRT-PCR.

The sensitivity of an assay for detection of blood-stage parasites, and thus the number of parasite positive individuals, can greatly influence the proportion of gametocyte carriers and therefore can explain differences between studies. The qPCR used in this study has a detection limit of one 18S rRNA gene copy/μL and detects even very low parasite densities typical for asymptomatic individuals. The same samples were screened with a nested PCR for *Pfmsp2* and *Pvmsp1*F3 followed by gel electrophoresis [40,41]. This assay was less sensitive and yielded lower parasite prevalence rates but higher proportions of gametocyte positive samples (*Pf*: 69.5%, *Pv*: 66.7%). In a different study, detection of parasites based on highly abundant RNA instead of DNA led to a pronounced increase in prevalence, but the proportion of gametocyte positive samples dropped from 59 to 41% for *P*. *falciparum* and from 53 to 36% for *P*. *vivax* [33]. In conclusion, the more sensitive the assay to detect blood-stage parasites, the more very low-density infections are detected, and the lower the proportion of gametocyte positive samples among all positive samples.

In this cross-sectional survey 56% *P*. *falciparum* and 81% *P*. *vivax* carriers had <100 18S rRNA gene copies/μL. The smaller proportion of *P*. *vivax* than *P*. *falciparum* gametocyte carriers is thus a consequence of the lower expression level of *pvs25* as compared to *pfs25* and the lower average densities of *P*. *vivax* infections.

As expected qRT-PCR estimates of gametocytaemia were much more closely linked to parasite densities by qPCR for *P*. *vivax* than for *P*. *falciparum*. *P*. *vivax* gametocytes mature rapidly [1] and follow levels of asexual parasitaemia [3]. Blood-stage parasitaemia is therefore a good surrogate marker for *P*. *vivax* gametocytaemia. *P*. *falciparum* gametocytes develop for 7–10 days in the bone marrow [42] before stage V gametocytes start appearing in the blood stream [1]. In *P*. *falciparum*, gametocytaemia is thus likely to be more related to the parasitaemia two weeks earlier than to concurrent parasitaemia.

LM and qRT-PCR estimates of gametocyte density were much more closely correlated for *P*. *falciparum* than *P*. *vivax*, possibly because the characteristic ‘banana-shaped’ *P*. *falciparum* gametocytes are more easily identifiable than *P*. *vivax* gametocytes, which resemble asexual parasites. At lower densities there was no correlation between the two measures for either species. By LM a maximum of 0.0625μL blood was counted making estimates of densities error-prone. Furthermore, *Plasmodium spp*. are probably not randomly distributed in blood, autoagglutination of asexual parasites was reported for *P*. *falciparum* [43]. It is possible that gametocytes also cluster to increase the chance that at least one male and one female gametocyte being taken up in a 2–5μL mosquito blood meal [44]. At densities <1 gametocyte/μL, detection of gametocytes in such small volumes of blood examined by LM becomes stochastic. Estimates of gametocytaemia by qRT-PCR based on RNA extracted from 50μL blood are thus likely more accurate than those by LM.

The contribution of very low-density gametocyte carriers to transmission is not clear. Infectiveness of individuals negative by microscopy has been repeatedly reported. *P*. *falciparum* infections with sub-patent gametocytaemia in children from Burkina Faso [45] and Kenya [27] infected 24.2% and 43.5% of *Anopheles gambiae*, mosquitoes, respectively. Similarly, the infectivity of sub-microscopic *P*. *vivax* infections has been shown in studies from Thailand [46–48], Sri Lanka [49], Peru [30] and PNG [19]. The qRT-PCR applied detects 1–5 gametocytes/50μL blood. At such low gametocyte densities, a mosquito might not always take up at least one male and one female gametocyte in its 3–5μL blood meal [44] and therefore not all qRT-PCR positive individuals might be infective. Further studies comparing gametocyte quantification by qRT-PCR to infectivity in mosquito feeding assays are thus needed to accurately quantify the contribution of these very low-density infections to malaria transmission.

## Supporting Information

S1 Fig Correlation between qPCR and LM estimates of *P*. *falciparum* and *P*. *vivax* parasite densities. The Spearman correlation for *P*. *falciparum* was 0.81 and for *P*. *vivax* 0.39.
(PDF)
