# Supplementary figures and images for: Blood-Stage Parasitaemia and Age Determine Plasmodium falciparum and P. vivax Gametocytaemia in Papua New Guinea
Source: PLoS One. 2015 May 21;10(5):e0126747. doi: 10.1371/journal.pone.0126747 (PMC4440770; doi:10.1371/journal.pone.0126747)

## *P. falciparum*

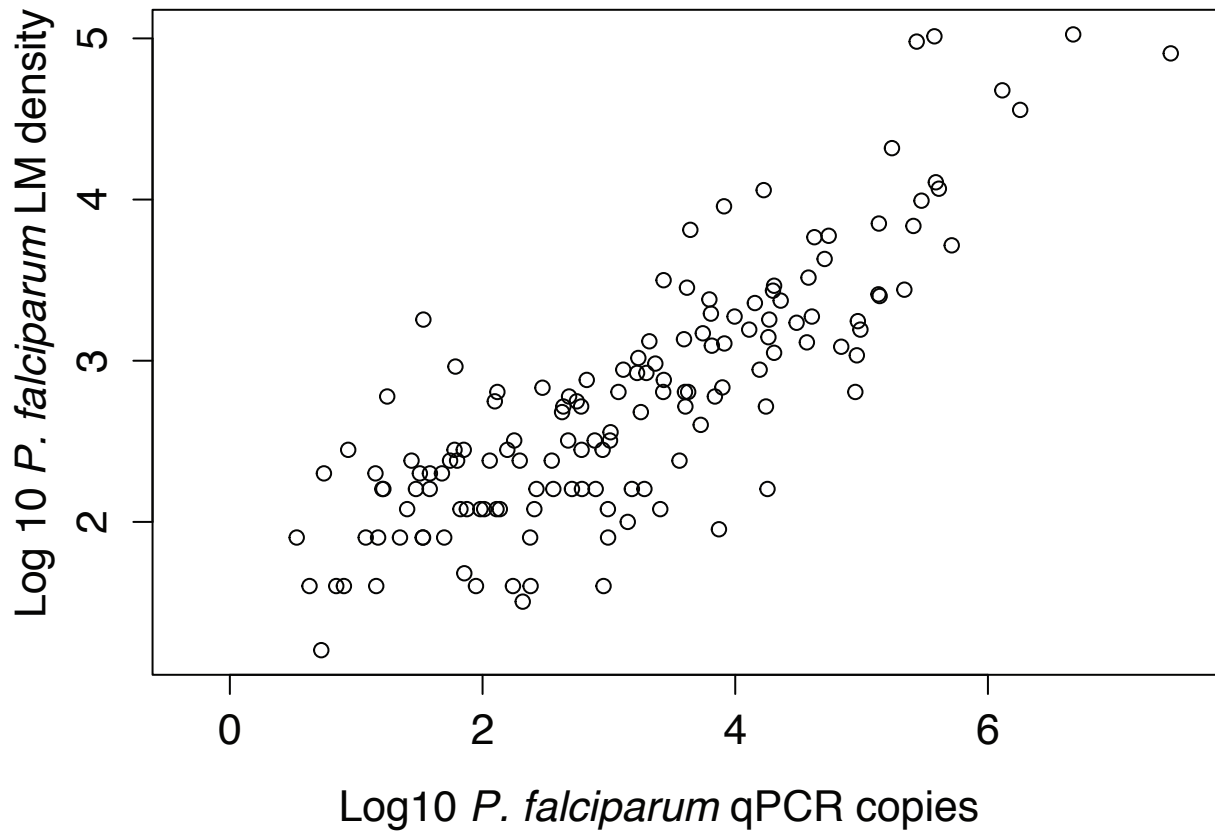

## *P. vivax*

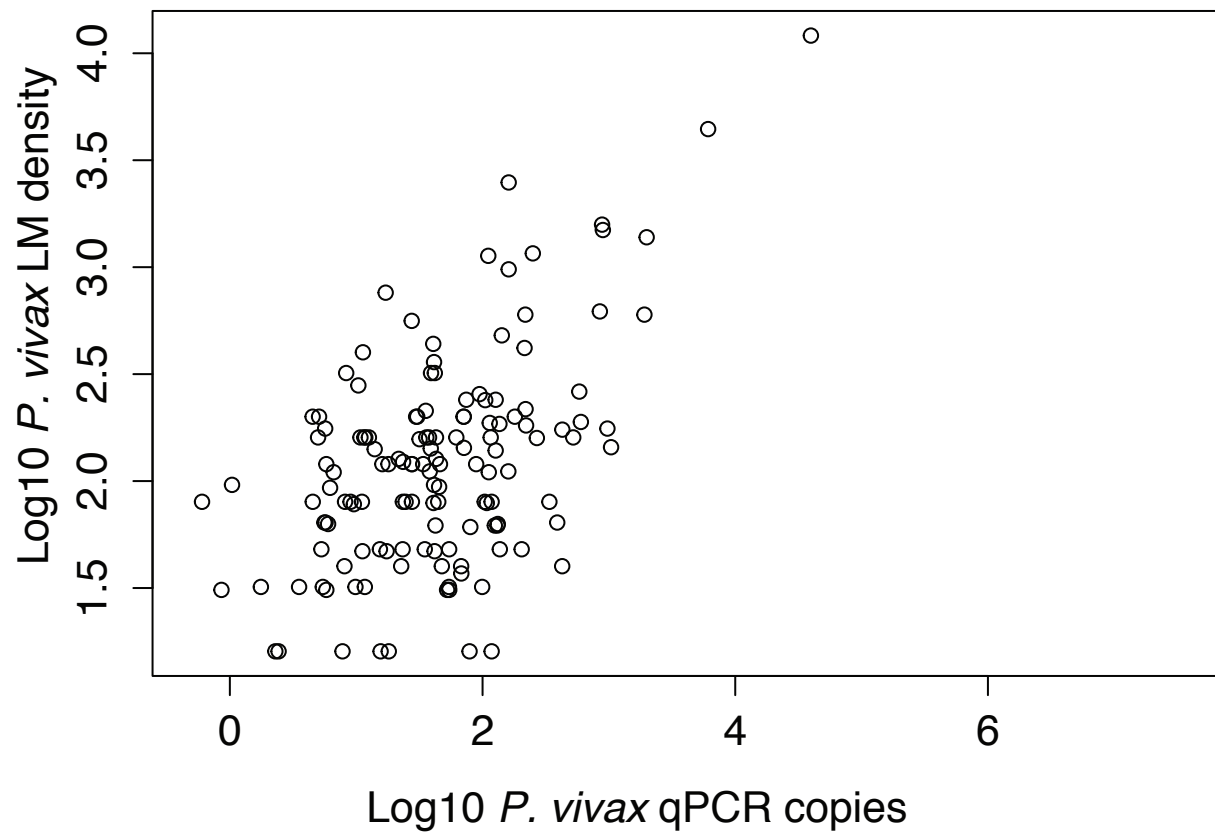

Supplement: S1 Fig — The Spearman correlation for P. falciparum was 0.81 and for P. vivax 0.39. (PDF) [file pone.0126747.s001.pdf]
